# The Role of Artificial Intelligence and Professional Expertise in Adapted Physical Activity Prescription for Orthopedic Rehabilitation

**DOI:** 10.3390/jfmk11010113

**Published:** 2026-03-09

**Authors:** Martina Sortino, Bruno Trovato, Rita Chiaramonte, Antonio Carrera, Marco Sapienza, Federico Roggio, Giuseppe Musumeci

**Affiliations:** 1 Department of Biomedical and Biotechnological Sciences, Section of Anatomy, Histology and Movement Science, School of Medicine, University of Catania, Via Santa Sofia 89, 95123 Catania, Italy; martinasortino97@gmail.com (M.S.); brunotrovato94@gmail.com (B.T.); antocarrera2000@gmail.com (A.C.); g.musumeci@unict.it (G.M.); 2 Physical Medicine and Rehabilitation Unit, AOU Policlinico G. Rodolico, University of Catania, 95123 Catania, Italy; ritachiaramd@gmail.com; 3 Department of General Surgery and Medical Surgical Specialties, Section of Orthopedics and Traumatology, AOU Policlinico-Vittorio Emanuele, University of Catania, 95123 Catania, Italy; marcosapienza09@yahoo.it; 4 Research Center on Motor Activities (CRAM), University of Catania, 95123 Catania, Italy; 5 Department of Biology, Sbarro Institute for Cancer Research and Molecular Medicine, College of Science and Technology, Temple University, Philadelphia, PA 19122, USA

**Keywords:** adapted physical activity, artificial intelligence, exercise protocol, muscoloskeletal disorders

## Abstract

**Background**: Adapted Physical Activity (APA) prescription is a complex decision-making process that integrates clinical guidelines and individual patient characteristics and remains strongly dependent on clinician experience. Generative artificial intelligence (AI) has recently emerged as a potential decision-support tool in exercise prescription; however, its interaction with professional expertise is still unclear. This study compared the perceived quality of APA protocols developed by expert professionals, novice professionals supported by AI, and AI operating autonomously across multiple orthopedic conditions. **Methods**: In this observational cross-sectional study, five real orthopedic prescriptions (scoliosis, low back pain, osteoporosis, high risk of falls, and osteoarthritis) were used to generate three APA protocols per condition: expert professional (EP), novice professional with AI support (NAI), and AI alone. All protocols were created using an identical standardized prompt and anonymized. A multidisciplinary panel of 135 professionals blindly evaluated the protocols using a structured questionnaire assessing effectiveness, safety, appropriateness, clarity, and progression. Overall quality scores were compared using Friedman tests with post hoc Wilcoxon signed-rank tests. **Results**: Across all conditions, EP protocols achieved the highest quality scores, followed by NAI, while AI-alone protocols consistently received the lowest ratings (all *p* < 0.05). NAI protocols showed intermediate performance, partially reducing the expertise gap. Post hoc analyses showed that EP protocols received significantly higher rating than AI protocols in all conditions (*p* < 0.01). NAI protocols received significantly higher rating than AI protocols in most conditions (*p* < 0.01) except osteoporosis (*p* = 0.362). Differences between EP and AI were most pronounced for safety (*p* < 0.01), appropriateness (tailoring *p* < 0.01), and progression (*p* < 0.05), whereas EP–NAI differences were smaller and condition-dependent. AI-alone protocols showed greater variability across pathologies. **Conclusions**: Professional expertise remains the main determinant of APA protocol quality. AI support can improve protocol structure and perceived quality when used by novice professionals but does not replace expert clinical reasoning. AI-generated protocols without human oversight are not yet suitable for autonomous APA prescription, supporting a complementary, expertise-dependent role of AI in exercise programming.

## 1. Introduction

Nowadays, physical activity and exercise are recognized worldwide as a fundamental tool to improve general well-being and health. The American College of Sport Medicine, one of the leading societies in the field of sports science, recommends the practice of exercise, sports, and physical activity to improve cardiorespiratory and neuromotor functions as well as musculoskeletal and mental health [1]. Adapted physical activity (APA) is a complex decision-making process, and it refers to all physical or sports activities tailored to the capacity and ability of patients with chronic disease, disability, aging, or any other condition that requires specific professional attention [2]. Evidence-based guidelines represent an essential reference for therapeutic exercise; however, in practice they primarily provide conceptual frameworks rather than directly applicable operational protocols. Their implementation requires case-specific adaptation, in which the professional interprets recommendations in light of the individual context [3].

Therefore, APA must account for pathology, comorbidities, functional limitations, and individual patient characteristics. Program design simultaneously involves load management, progression, safety, and adherence, making protocol quality highly dependent on the professional’s experience and clinical reasoning abilities [4,5]. The topic of inter-rater evaluation and program development is widely studied in the literature. Professionals with comparable level of experience, especially at early-career stages, may produce different evaluations or exercise programs for the same individual given identical information [6,7]. In line with established skill-acquisition frameworks (e.g., Dreyfus/Benner), novices tend to rely more on rule-based reasoning and have less situational pattern recognition than experienced practitioners, which can contribute to greater variability in decision-making [8]. This issue becomes greater when professionals with different levels of experience are involved; given identical input, they may produce qualitatively different protocols or evaluations [9]. The interpretative requirement and the great amount of information to consider when creating tailored APA programs contributes to introduce inter-rater variability in exercise programming between professionals. Clinical expertise allows for greater structuring of reasoning and more targeted stimulus selection, whereas programming by less experienced professionals may be more fragmented or less systematic, with potential repercussions on intervention effectiveness [10].

Within this context, artificial intelligence (AI), especially generative language models, is emerging as a decision-support tool in complex planning processes. In recent years, artificial intelligence has become more powerful and more employed in a wide variety of scientific fields, entering also the realm of general health, sport medicine, physiotherapy, and kinesiology [11,12]. The use of AI should not be intended to replace the professional, but rather to support the clinician’s decision-making by facilitating the structuring of the APA programs, and the retrieval of scientific evidence, accelerating the process. These characteristics suggest a potential role for AI in reducing decision-making variability, particularly beneficial for less experienced professionals [13,14]. Despite growing interest in the application of AI within exercise sciences, there is a lack of studies systematically examining the interaction between professional expertise and AI use in APA prescription. Specifically, it remains unclear whether AI support differentially influences protocol quality between expert and novice professionals, or whether it can contribute to reducing experience-related performance gaps. It is important to underline that generative language models may exhibit sycophancy with the user [15], which could be particularly problematic when developing APA programs for clinical populations.

Thus, the aim of this study is to compare the quality of protocols produced by health professionals with different levels of expertise, with and without AI support, as well as protocols generated autonomously by AI, through a blinded, multidisciplinary evaluation based on criteria of coherence, safety, progression, clarity, and load appropriateness.

## 2. Materials and Methods

This observational cross-sectional study evaluated the perceived quality of APA protocols designed in response to orthopedic prescriptions, using a blinded expert-based assessment framework. The study aimed to compare three different protocol-design approaches: expert human professionals (EP), novice professionals supported by artificial intelligence (NAI), and artificial intelligence (AI) operating autonomously. The study did not involve patients or clinical interventions and was based exclusively on anonymized clinical prescriptions and anonymous questionnaire-based evaluations conducted by healthcare and movement science professionals. The study was conducted at the Research Center on Motor Activities (CRAM), University of Catania.

An orthopedic physician provided five real clinical prescriptions, which were fully anonymized prior to analysis. The prescriptions referred to patients with the following conditions: scoliosis, lower-limb osteoarthritis, osteoporosis, high risk of falls, and low back pain. With different outcomes requests, all prescriptions explicitly required the implementation of a long-term postural exercise program. These prescriptions served as the clinical input for the development of the APA protocols. The study protocol was approved by the Scientific Committee of the Research Center on Motor Activities (CRAM), University of Catania (protocol no. CRAM-55-2024, 17 July 2024), and all procedures were conducted in accordance with the Declaration of Helsinki. Participation in the evaluation process was voluntary and anonymous, and completion of the questionnaire implied informed consent.

### 2.1. Characteristics of the APA Protocols

For each prescription, three distinct APA protocols were developed under the following scenarios:

(1) Protocol designed by an experienced kinesiologist (EP) with >10 years’ experience in postural assessment and postural exercise, holding an MSc, a postgraduate specialization, and a PhD, in the field of preventive and adapted exercise activities;
(2) Protocol designed by a novice kinesiologist (NAI; <2 years’ experience), holding an MSc in Preventive and Adapted Physical Activity, and supported by a generative AI model (GPT-5);
(3) Protocol designed exclusively by the generative AI model (GPT-5), without human intervention (AI).

To ensure methodological consistency across the three protocol-design scenarios, the same standardized prompt was provided to all conditions. The prompt required the development of APA programs was structured as follows:

*“Design a mesocycle of Adapted Physical Activity/postural exercise for the following subject: (subject description). Develop the program based on the request of the orthopedic physician: (orthopedic prescription). Prepare the protocol allowing for, but not limited to, the use of exercise equipment. Consider that this represents the first mesocycle of training, that each session must last 60 min, and that the weekly frequency must be two sessions per week. Clearly specify exercises, sets, repetitions (or execution time), and progression criteria.”*

No additional guidance, examples, or corrective feedback was provided beyond the content of the prompt. This approach was adopted to isolate the effect of the protocol-design strategy and to ensure comparability across the three development scenarios. All protocols were structured over a one-month period with two sessions per week. Each protocol included exercise descriptions, intended functional and postural objectives, progression criteria, and general precautions. Following development, all protocols were anonymized and coded to ensure blind evaluation.

### 2.2. Evaluation Procedure

A custom questionnaire was developed using Google Forms to assess the perceived quality of the APA protocols. The questionnaire consisted of six items evaluating the following domains: perceived effectiveness, safety, clinical appropriateness, clarity and ease of understanding, and progression structure. Each item was rated on a 5-point Likert scale ranging from 1 to 5, with higher scores indicating more favorable evaluations (Supplementary S1).

A total of 135 professionals participated in the evaluation process, including medical doctors, orthopedic specialists, physiatrists, physiotherapists, kinesiologists, and APA professionals. For each clinical condition, evaluators assigned to assess a specific pathology independently reviewed all three APA protocols developed for that same condition (EP, NAI, AI). Thus, each participant performed a within-pathology comparative evaluation, ensuring that the three protocol-design approaches were judged under identical clinical assumptions.

**Figure 1:**
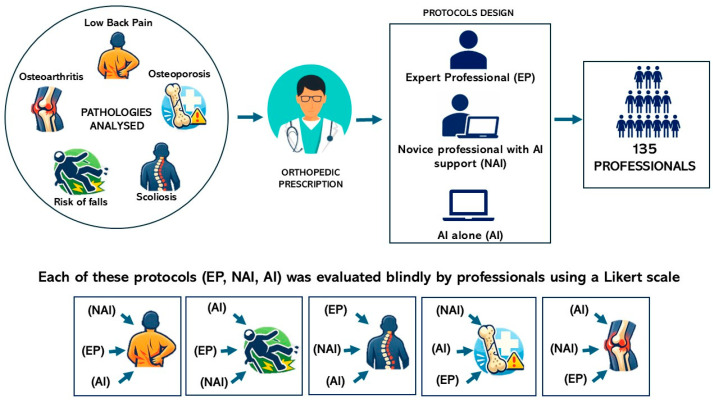
Overview of the study workflow used to compare APA protocol design approaches.

To minimize potential order effects and evaluation bias, the presentation order of the three protocols was randomized within the Google Forms questionnaire for each pathology. All evaluations were conducted in a fully blinded manner. Participants were not informed about the authorship of the protocols nor about the involvement of artificial intelligence in their development. Participation was voluntary and anonymous, they reported only their profession and their years of experience. Study framework is shown in Figure 1.

### 2.3. Statistical Analysis

All statistical analyses were performed with R Studio (version 4.5.2). The five evaluation domains (perceived effectiveness, safety, appropriateness, clarity of indications, and progression structure) were coded as numeric Likert responses (0–5). For each participant and for each of the three protocol conditions, an overall protocol score was computed as the mean of the five domain ratings (i.e., the row-wise average across the five items). Given the ordinal nature of Likert-type outcomes and the within-subject design, between-condition comparisons were conducted using the Friedman test. When the Friedman test indicated significant differences, post hoc pairwise comparisons were performed using paired Wilcoxon signed-rank tests with Bonferroni correction for multiple comparisons. For each pathology and protocol condition, descriptive statistics were reported as mean and standard deviation of the overall protocol score. The statistical significance threshold was set at *p* < 0.05.

## 3. Results

A total of 135 professionals completed the evaluation process. The sample included experts from different healthcare and movement science backgrounds with heterogeneous levels of professional experience from the same country. Specifically, 47 kinesiologists participated in the study, with a mean professional experience of 10.8 years. A total of 15 physiotherapists contributed to the evaluation, reporting a higher mean experience of 15.6 years. The medical group consisted of 56 fully qualified physicians, with a mean professional experience of 8.5 years, and 17 medical residents, who reported a mean experience of 2.1 years.

Across all investigated pathologies, the APA protocols designed by the EP consistently achieved the highest overall evaluation scores, followed by the protocols developed by the NAI, while the AI protocols received the lowest scores. Mean values were generally high across conditions, indicating a favorable overall perception of protocol quality, Table 1. Expert-designed protocols consistently demonstrated higher mean scores and lower variability across all pathologies compared to AI protocols, while NAI co-designed protocols generally showed intermediate values.

**Figure 2:**
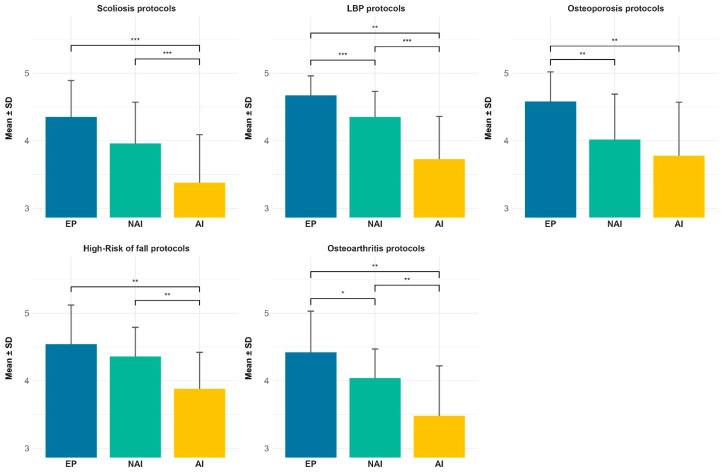
Differences in perceived APA protocol quality across EP (expert professional), NAI (novice professional + AI support), and AI (artificial intelligence) alone; * *p* < 0.05; ** *p* < 0.01; *** *p* < 0.001.

For each pathology, non-parametric Friedman tests revealed statistically significant differences among the three protocol-design conditions (all *p* < 0.001). Post hoc pairwise comparisons identified significant differences between AI, EP and NAI protocols across all conditions, Table 2. Graphical comparison between the three protocols is reported in Figure 2.

**Figure 3:**
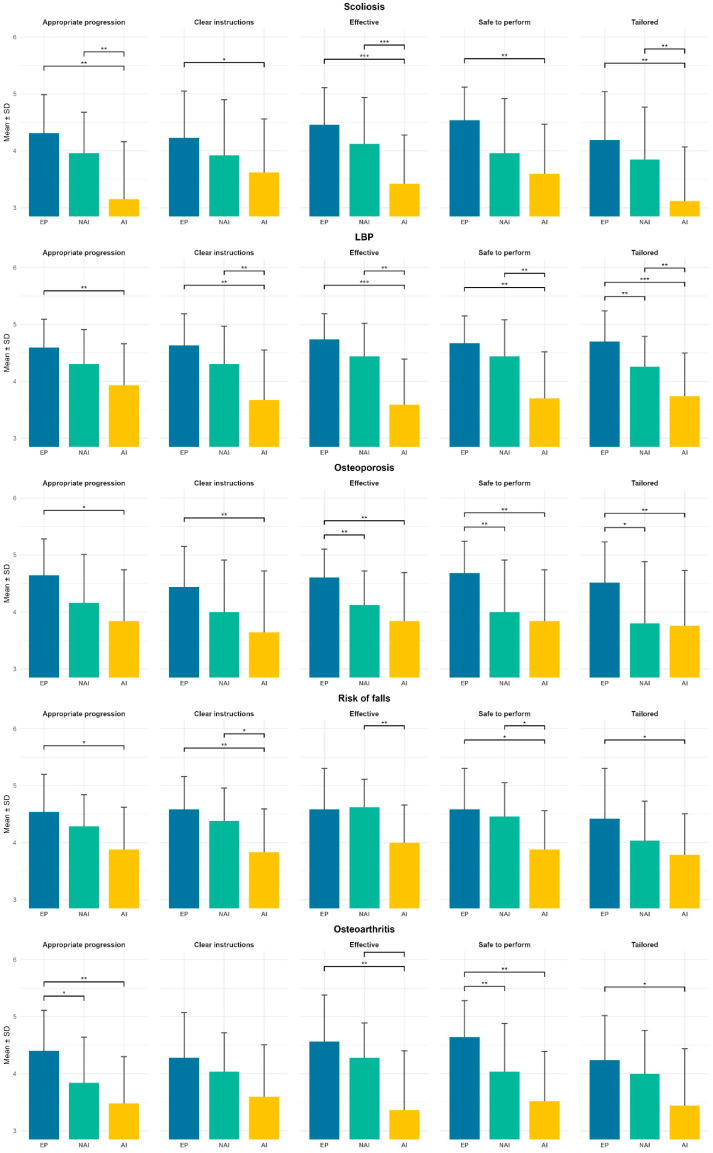
Perceived Quality of APA Protocols for the specific items of the questionnaire; * *p* < 0.05; ** *p* < 0.01; *** *p* < 0.001.

Concerning the specific questionnaire items-analysis, EP protocols consistently received higher perceived quality scores than those developed by NAI and by AI. Overall differences among protocols were significant for most quality items according to the Friedman test (*p* < 0.05), Table 3 and Figure 3. In scoliosis and osteoporosis, post hoc analyses showed significant differences mainly between EP and AI, while EP–NAI and NAI–AI comparisons were often non-significant. For LBP, clearer separations emerged, with EP protocols significantly outperforming AI across all items and several significant differences also observed between NAI and AI. In the high risk of falls condition, perceived quality scores were generally high across protocols, with limited differences between EP and NAI but more frequent differences involving AI, particularly for clarity and safety. In lower-limb osteoarthritis, EP protocols were rated significantly higher than AI for most items, whereas EP–NAI differences were minimal.

## 4. Discussion

The present study analyzed differences in the quality of APA protocols developed by: (i) an expert clinician, (ii) a novice clinician supported by an AI model, and (iii) an AI model operating independently. Based on exercise prescriptions provided by an orthopedic specialist for five common orthopedic conditions, namely scoliosis, low back pain, osteoporosis, high risk of falls, and lower-limb osteoarthritis, the involved actors developed condition-specific APA protocols. These protocols were then blindly evaluated by a panel of field experts, including medical doctors, personal trainers, kinesiologists, and physiotherapists. Overall, the findings indicate that professional expertise remains a key determinant of programming quality. While AI support was able to modify specific qualitative aspects of the protocols developed by the less experienced professional, the resulting outputs remained clearly distinct from those produced by an expert clinician, particularly in dimensions requiring advanced clinical reasoning.

Previous research suggests that clinicians and exercise professionals tend to evaluate protocol quality according to core criteria such as provider characteristics, delivery modality, setting, dosage, tailoring, and adherence, as highlighted by the Delphi study of Slade et al. [16]. In the present study, protocols developed by the expert professional consistently achieved higher quality ratings, reflecting a stronger alignment with these criteria compared with those produced by the novice professional, even when AI support was provided. This finding reinforces the central role of clinical experience in structuring coherent exercise prescriptions, integrating scientific evidence, and managing training load appropriately. Such results are consistent with theoretical models of clinical reasoning, which posit that expertise enables more efficient integration of relevant information, more appropriate stimulus selection, and greater contextualization of interventions, all of which are essential for ensuring safety and minimizing clinical risk [17]. Although AI support reduced the quality gap between novice- and expert-developed protocols, it did not eliminate it.

More specifically, AI appeared to function as a compensatory tool for novice professionals by enhancing structural coherence and organization of the protocols, while also substantially accelerating the drafting process, as previously reported in the literature [18]. This aligns with evidence suggesting that generative language models can improve writing efficiency and, when used as supportive tools, may assist clinicians in specific decision-making tasks [19]. However, despite these advantages, AI-generated content showed limitations in clinical reasoning, often producing programs that were overly generic and insufficiently tailored to the specific medical prescriptions provided.

Based on these findings, it may be hypothesized that AI can support novice professionals during the decision-making phases of exercise prescription by offering a structuring framework that reduces the fragmentation typically observed in early stages of clinical practice [20,21,22]. In the present study, AI was not used by the expert clinician, limiting direct conclusions regarding its potential role in expert-driven programming. Nonetheless, drawing on the observed effects in novice professionals and on established models of clinical expertise, it is reasonable to speculate that AI could serve a different function for experienced clinicians. Rather than acting as a structuring guide, AI may support experts as a metacognitive tool, facilitating systematic comparison with current evidence and guidelines without replacing professional judgment. This interpretation is consistent with the notion that increasing expertise does not negate the utility of AI, but rather reshapes its role from organizational support to cognitive refinement [23].

Across clinical conditions, a consistent gap was observed between expert-developed and AI-generated protocols, particularly for items related to safety, appropriate progression, and effectiveness. In scoliosis, the largest differences concerned progression, effectiveness, specificity, and safety, in line with SOSORT recommendations emphasizing individualized and condition-specific exercise approaches, particularly during growth, where careful tailoring to the patient and clinical context is essential [24]. These features may be challenging for generic AI-generated prescriptions to fully capture.

For low back pain, discrepancies were most pronounced for specificity and effectiveness, likely reflecting the importance of subgroup-based, progressively dosed interventions emphasized in current guidelines [25]. In osteoporosis, differences were especially evident in safety and effectiveness, consistent with consensus statements highlighting the need for carefully dosed loading strategies that maximize musculoskeletal benefits while minimizing fracture risk [26,27]. Such considerations require nuanced clinical judgment, which may currently exceed AI capabilities. Notably, the high-risk-of-falls protocols showed the smallest differences between expert and AI outputs, possibly because fall-prevention exercise programs are often highly standardized and consensus-driven [28], making them easier for AI to reproduce in a guideline-consistent manner. In contrast, for osteoarthritis, substantial gaps between expert and AI protocols were again evident for safety, progression, and effectiveness, reflecting international recommendations that emphasize appropriate dosing, graded progression, and symptom-responsive modifications as essential components of first-line therapeutic exercise [29]. Collectively, these findings align with broader evidence indicating that AI-generated exercise prescriptions may appear plausible but remain incomplete [30], and that current large language models are not yet suitable for autonomous clinical decision-making. This reinforces the importance of expert oversight when AI is integrated into clinical exercise prescription workflows [31].

An additional relevant observation is that expert-developed protocols exhibited lower variability in quality scores across all conditions, whereas novice- and AI-generated outputs showed greater variability between pathologies. This pattern is consistent with previous studies demonstrating that expert clinicians are better able to maintain consistently high-quality standards across different clinical scenarios [32,33]. The multidisciplinary composition of the evaluation panel represents a further strength of the study, as it reflects the collaborative nature of APA practice and enhances the robustness of protocol assessment. The results suggest that AI can enhance programming quality in novice clinicians, provided it is used critically and without uncritical acceptance of generated outputs.

Overall, the findings point to a dynamic interaction between professional expertise and AI use. For less experienced professionals, AI may facilitate the development of more structured and systematic decision-making processes, whereas for expert clinicians it may serve as a supportive resource for evidence consultation rather than a substitute for clinical reasoning.

Several limitations should be acknowledged. The number of clinical cases was limited, and exercise prescriptions were developed without iterative feedback from the prescribing orthopedic specialist. In addition, the study did not include a novice-only control group. While our comparisons (EP, AI, and NAI) allow us to examine differences between EP and AI programs, we cannot determine how much AI improves performance compared with novices working without AI. Moreover, AI protocol development was restricted to a one-shot interaction (single standardized prompt without iterative refinement). In real-world settings, iterative prompting could yield different outputs; therefore, generalizability to routine clinical workflows should be interpreted with caution. The absence of real-world implementation and clinical outcomes prevents determination of whether the observed differences in perceived quality translate into meaningful differences in effectiveness, safety, or adherence. Future studies should investigate the impact of AI-supported exercise prescription in applied clinical settings and explore how AI tools may be integrated into expert-led APA programming without compromising professional autonomy or decision-making quality.

## 5. Conclusions

This study shows that professional expertise remains the main determinant of APA protocol quality. Expert-designed programs consistently outperformed both AI-assisted and fully AI-generated protocols, particularly in safety, progression, and effectiveness. AI support improved the structural quality of protocols developed by novice professionals but did not eliminate the gap with expert-driven programs, confirming that current generative models are not suitable for autonomous clinical exercise prescription. Overall, AI appears to be a useful decision-support tool for less experienced professionals, while expert oversight remains essential. Future research should evaluate AI-assisted APA programming in real clinical settings and explore its integration into expert-led practice without compromising clinical judgment or safety.

## Figures and Tables

**Table 1:** Overall mean and SD of evaluation scores for the three protocol-design approaches.

Pathology	EP	NAI	AI	*p*-Value ^+^
Scoliosis	4.35 (0.54)	3.96 (0.61)	3.38 (0.71)	<0.001
Low back pain (LBP)	4.67 (0.29)	4.35 (0.38)	3.73 (0.63)	<0.001
Osteoporosis	4.58 (0.44)	4.02 (0.67)	3.78 (0.79)	0.002
High risk of falls	4.54 (0.58)	4.36 (0.43)	3.88 (0.54)	<0.001
Osteoarthritis	4.42 (0.61)	4.04 (0.43)	3.48 (0.74)	<0.001

^+^ according to Friedmann test; EP: expert human professionals; NAI: novice professionals, AI: artificial intelligence.

**Table 2:** Post hoc pairwise comparisons.

Pathology	EP vs. NAI	EP vs. AI	NAI vs. AI
Scoliosis	*p* = 0.051	*p* < 0.001	*p* < 0.001
Low back pain (LBP)	*p* < 0.001	*p* = 0.002	*p* < 0.001
Osteoporosis	*p* = 0.001	*p* = 0.001	*p* = 0.362
High risk of falls	*p* = 0.444	*p* = 0.006	*p* = 0.002
Osteoarthritis	*p* = 0.012	*p* = 0.001	*p* = 0.001

EP: expert human professionals; NAI: novice professionals, AI: artificial intelligence.

**Table 3:** Descriptive statistics of perceived program quality items by protocol.

	Items	EP Mean ± SD	NAI Mean ± SD	AI Mean ± SD	*p*-Value ^+^
Scoliosis	Tailored	4.19 (0.85)	3.85 (0.92)	3.12 (0.95)	<0.001
	Effective	4.46 (0.65)	4.12 (0.82)	3.42 (0.86)	<0.001
	Clear instructions	4.23 (0.82)	3.92 (0.98)	3.62 (0.94)	<0.001
	Appropriate progression	4.31 (0.68)	3.96 (0.72)	3.15 (1.01)	0.019
	Safe to perform	4.54 (0.58)	3.96 (0.96)	3.6 (0.87)	<0.001
LBP	Tailored	4.7 (0.54)	4.26 (0.53)	3.74 (0.76)	<0.001
	Effective	4.74 (0.45)	4.44 (0.58)	3.59 (0.8)	<0.001
	Clear instructions	4.63 (0.56)	4.3 (0.67)	3.67 (0.88)	<0.001
	Appropriate progression	4.59 (0.5)	4.3 (0.61)	3.93 (0.73)	0.001
	Safe to perform	4.67 (0.48)	4.44 (0.64)	3.7 (0.82)	<0.001
Osteoporosis	Tailored	4.52 (0.71)	3.8 (1.08)	3.76 (0.97)	<0.001
	Effective	4.6 (0.5)	4.12 (0.6)	3.84 (0.85)	<0.001
	Clear instructions	4.44 (0.71)	4 (0.91)	3.64 (1.08)	0.003
	Appropriate progression	4.64 (0.64)	4.16 (0.85)	3.84 (0.9)	0.003
	Safe to perform	4.68 (0.56)	4 (0.91)	3.84 (0.9)	0.003
Risk of falls	Tailored	4.42 (0.88)	4.04 (0.69)	3.79 (0.72)	0.001
	Effective	4.58 (0.72)	4.62 (0.49)	4 (0.66)	0.002
	Clear instructions	4.58 (0.58)	4.38 (0.58)	3.83 (0.76)	0.004
	Appropriate progression	4.54 (0.66)	4.29 (0.55)	3.88 (0.74)	<0.001
	Safe to perform	4.58 (0.72)	4.46 (0.59)	3.88 (0.68)	0.005
Osteoarthritis	Tailored	4.24 (0.78)	4 (0.76)	3.44 (1)	0.002
	Effective	4.56 (0.82)	4.28 (0.61)	3.36 (1.04)	<0.001
	Clear instructions	4.28 (0.79)	4.04 (0.68)	3.6 (0.91)	0.011
	Appropriate progression	4.4 (0.71)	3.84 (0.8)	3.48 (0.82)	0.021
	Safe to perform	4.64 (0.64)	4.04 (0.84)	3.52 (0.87)	0.002

^+^ according to Friedmann test; EP: expert human professionals; NAI: novice professionals, AI: artificial intelligence.

## Data Availability

The original contributions presented in this study are included in the article/Supplementary Materials. Further inquiries can be directed to the corresponding author.

## Supplementary Materials

The following supporting information can be downloaded at: https://www.mdpi.com/article/10.3390/jfmk11010113/s1, Table S1: Expert evaluation questionnaire for the proposed exercise protocols.
