# Supplementary material for: The Role of Artificial Intelligence and Professional Expertise in Adapted Physical Activity Prescription for Orthopedic Rehabilitation
Source: J Funct Morphol Kinesiol. 2026 Mar 9;11(1):113. doi: 10.3390/jfmk11010113 (PMC13027591; doi:10.3390/jfmk11010113)
Supplement: Supplementary file 1 [file jfmk-11-00113-s001.zip › jfmk-4166443-supplementary.pdf]

CASE [PATHOLOGY]

Before starting, please enter your details:
(* indicates a required question)
Name (e.g., "A. C."):*
Profession:*
Years of experience:*

Each of the three protocols represents a work mesocycle, structured into **two weekly sessions**, each lasting a **maximum of 60 minutes**.

Clinical history (Anamnesis)

Clinical history was individualized according to the specific pathology represented in each case; therefore, the input clinical profile varied across the included conditions.

Clinical / exercise prescription

The prescription was individualized according to the participant's specific condition; consequently, the recommended program varied across the included pathologies.

Protocol no. 1 (click the link to view)

[link to the pdf]

Evaluation questions

How effective do you think the protocol could be in improving the participant's postural/clinical condition?

1
2
3
4
5

Is the protocol safe for the participant's condition and does it respect the principles of gradual progression?

1
2
3
4
5

Is the protocol adapted to the participant's characteristics (e.g., age, disease severity, activity level)?

1
2
3
4
5

Are the instructions easy to understand and replicate in a clinical setting?

1
2
3
4
5

Is the protocol sustainable over time?

1
2
3
4
5

Comment (optional)

[free text]

Protocol no. 2 (click the link to view)

[link to the pdf]

Evaluation questions

How effective do you think the protocol could be in improving the participant's postural/clinical condition?

1
2
3
4
5

Is the protocol safe for the participant's condition and does it respect the principles of gradual progression?

1
2
3
4
5

Is the protocol adapted to the participant's characteristics (e.g., age, disease severity, activity level)?

1
2
3
4
5

Are the instructions easy to understand and replicate in a clinical setting?

1
2
3
4
5

Is the protocol sustainable over time?

1
2
3
4
5

Comment (optional)

[free text]

Protocol no. 3 (click the link to view)

[link to the pdf]

Evaluation questions

How effective do you think the protocol could be in improving the participant's postural/clinical condition?

1
2
3
4
5

Is the protocol safe for the participant's condition and does it respect the principles of gradual progression?

1
2
3
4
5

Is the protocol adapted to the participant's characteristics (e.g., age, disease severity, activity level)?

1
2
3
4
5

Are the instructions easy to understand and replicate in a clinical setting?

1
2
3
4
5

Is the protocol sustainable over time?

1
2
3
4
5

Comment (optional)

[free text]
